# Paradoxical Roles of the Neutrophil in Sepsis: Protective and Deleterious

**DOI:** 10.3389/fimmu.2016.00155

**Published:** 2016-04-26

**Authors:** Fabiane Sônego, Fernanda Vargas e Silva Castanheira, Raphael Gomes Ferreira, Alexandre Kanashiro, Caio Abner Vitorino Gonçalves Leite, Daniele Carvalho Nascimento, David Fernando Colón, Vanessa de Fátima Borges, José Carlos Alves-Filho, Fernando Queiróz Cunha

**Affiliations:** ^1^Departamento de Farmacologia, Faculdade de Medicina de Ribeirão Preto, Universidade de São Paulo, Ribeirão Preto, Brazil; ^2^Departamento de Bioquímica e Imunologia, Faculdade de Medicina de Ribeirão Preto, Universidade de São Paulo, Ribeirão Preto, Brazil

**Keywords:** sepsis, neutrophil migration, organ dysfunction, toll-like receptors, chemotactic receptors

## Abstract

Sepsis, an overwhelming inflammatory response syndrome secondary to infection, is one of the costliest and deadliest medical conditions worldwide. Neutrophils are classically considered to be essential players in the host defense against invading pathogens. However, several investigations have shown that impairment of neutrophil migration to the site of infection, also referred to as neutrophil paralysis, occurs during severe sepsis, resulting in an inability of the host to contain and eliminate the infection. On the other hand, the neutrophil antibacterial arsenal contributes to tissue damage and the development of organ dysfunction during sepsis. In this review, we provide an overview of the main events in which neutrophils play a beneficial or deleterious role in the outcome of sepsis.

## Introduction

Sepsis represents a challenging health care and economical problem worldwide with lingering aftereffects (1). The incidence of sepsis has increased over the last decades (2). In terms of diagnosis, sepsis is a systemic response to infection, with increasing severity recognized as severe sepsis or septic shock. Severe sepsis is defined as sepsis in the presence of organ dysfunction and septic shock as the presence of hypotension unresponsive to vasoconstrictors (3). Intensive preclinical studies performed in the last decades have contributed greatly to the understanding the pathophysiology of sepsis, though it is not yet fully understood. Neutrophils are important players in the outcome of sepsis. Therefore, we will review the involvement of neutrophils in the pathophysiology of sepsis in this work.

## Control of Infections by Neutrophils

Neutrophils are leukocytes with multi-lobed nuclei that form in the bone marrow and are released in their mature form to the blood. Neutrophils have a short life span and do not show proliferative properties (4, 5).

Classically recognized as phagocytic cells, neutrophils are associated with the innate immune response. These cells are recruited to the site of the infection in response to chemotactic mediators, where they play antimicrobial roles (5, 6).

The presence of neutrophils at the site of infection has been demonstrated to be essential for controlling the bacterial and fungal burden and avoiding the systemic spread of the infection (7). Indeed, depletion of neutrophils in mice infected with *Staphylococcus aureus* markedly reduced the clearance of the bacteria and also survival (8). Similarly, depletion of neutrophils in mice infected with *Candida albicans* induced dissemination of the fungus and led to a higher mortality rate. Likewise, neutropenic patients are more susceptible to bacterial and fungal infections (9–11).

Neutrophils induce killing of pathogens *via* phagocytosis, degranulation, or even the release of intracellular components such as DNA, histones, and lytic proteins, which form neutrophil extracellular traps (NETs) (12, 13). Nitric oxide (NO), a mediator produced by the enzyme inducible nitric oxide synthase (iNOS), is one crucial mediator of the microbicidal activity of neutrophils. Deletion of *iNOS* induces a high mortality rate due to impaired control of the infection, despite the presence of neutrophils in the locale of the infection (14).

Additionally, neutrophils are equipped with receptors that recognize pathogen-associated molecular patterns or damage-associated molecular patterns, initiating signaling cascades and leading to the production of inflammatory mediators to establish an appropriate response against the pathogen. This results in amplification of the inflammatory process, including emigration of the new waves of neutrophils to the site of infection (15).

Chemokines are a family of small cytokines that are divided into small subfamilies based on variations of a conserved cysteine motif and play an important role in neutrophil recruitment (16). Most chemokines belong to the CC and CXC chemokine subfamilies (17), which exhibit two juxtaposed cysteine residues or one amino acid between the first two cysteine residues, respectively (18). Under physiological conditions, lymphocytes, monocytes, and macrophages express CC receptors (CCR) and respond to CC chemokines, whereas neutrophils express CXC receptors (CXCR)1 (IL-8R in humans) and CXCR2 and respond to CXC chemokines (19).

## Neutrophil Migration is Impaired during Severe Sepsis

As mentioned above, the control of an infection depends on the efficient migration of neutrophils to the site of infection as well as appropriate microbicidal activity (20). Our group and others have demonstrated that mice subjected to severe sepsis show inadequate migration of neutrophils to the site of infection, despite the high levels of chemokines at the site. The insufficient number of neutrophils recruited to the site of infection does not control the infection locally, contributing to the systemic spread of the pathogen. As consequence, a marked systemic inflammatory response is established, which is associated with high mortality rates (21).

Among the mechanisms leading to the failure of neutrophil migration, it has been shown that CXCR2 is internalized in circulating neutrophils from mice or patients with severe sepsis (22–24). Accordingly, neutrophils isolated from septic patients show reduced migration toward chemotactic mediators *ex vivo*, which is associated with patient survival: survivors show higher neutrophil migration compared with non-survivors (25).

**Figure 1:**
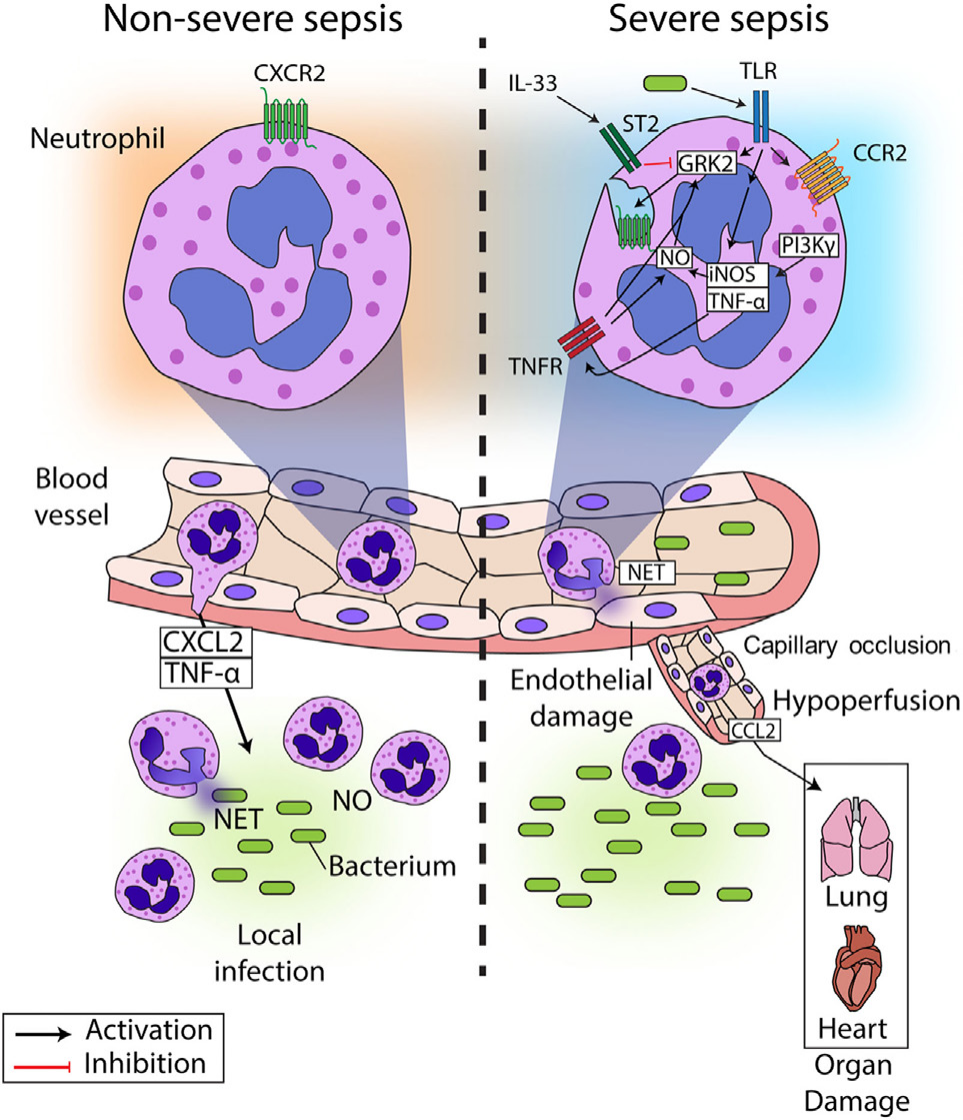
**Neutrophil migration in sepsis**. During non-severe sepsis, neutrophils expressing CXCR2 are recruited from the blood to the site of infection in response to CXCL2 and other chemoattractants. Neutrophils migrate to the locale of the infection, where they release NETs and produce reactive oxygen and nitrogen intermediates (such as NO) to kill the pathogens and avoid its spreading. By contrast, neutrophils are systemically stimulated during severe sepsis, which leads to impaired neutrophil migration to the infection focus. Bacterial components present in the blood activate TLRs expressed on neutrophils, leading to the up-regulation of GRK2, which induces desensitization of CXCR2 on the neutrophil surface. Additionally, TLR activation induces the expression of TNF-α and iNOS, the latter of which might also be activated by PI3K. Both TNF-α and NO can lead to upregulation of GRK2, exacerbating the down-regulation of CXCR2 on the neutrophil surface. As a consequence, neutrophil migration fails, and bacterial growth is not controlled. Furthermore, activation of TLRs also induces the expression of CCR2 on the surface of neutrophils, favoring the recruitment of these cells to distant organs producing CCL2, which contributes to organ damage in association with the capillary occlusion and the hypoperfusion observed in sepsis. The systemic activation of neutrophils also induces the release of NETs in the blood vessels, which causes endothelial damage, culminating in the aggravation of sepsis and possible death. Interestingly, it has been demonstrated that IL-33 can prevent the upregulation of GRK2 expression induced by TLR overactivation and consequently prevent the failure of neutrophil migration to the site of infection.

In recent years, several studies have described the mechanisms underlying CXCR2 internalization in circulating neutrophils during sepsis, resulting in failure of migration to the infectious focus (Figure 1) (24, 26–29). It has been reported that chemokine receptors belong to the G protein-coupled receptors (GPCRs), and their expression is precisely regulated (30). Prolonged or repeated exposure to agonists induces desensitization and internalization of GPCRs in a process dependent on the activation of GPCR kinases (GRKs) (31). GRKs phosphorylate the intracellular domains of the activated GPCR, leading to the recruitment of arrestin, which decouples the G protein from the receptor and trigger its internalization (32). Our group and others have demonstrated that ligands of Toll-like receptor (TLR)2, TLR4, and TLR9 (lipoteichoic acid (LTA), lipopolysaccharide (LPS), and CpG-oligodeoxynucleotide, respectively) induce GRK2 upregulation in circulating neutrophils, which in turn, leads to CXCR2 internalization (26, 29, 33–35). Indeed, pretreatment of neutrophils with a GRK2 inhibitor prevented the effect of TLR4 and TLR9 activation on CXCR2 internalization (33, 34). Corroborating these data, *tlr*2-, 4-, and 9-deficient mice show an increase in CXCR2 expression on circulating neutrophils, compared with WT mice subjected to severe sepsis (26, 34, 36). Additionally, IL-33, a member of the IL-1 family that binds to the heterodimeric receptor complex ST2, has been shown to prevent the up-regulation of GRK2 mediated by TLR signaling. IL-33 treatment improved the recruitment of neutrophils to the site of infection in mice and prevented LPS-induced chemotaxis reduction in human neutrophils (33, 37).

It is noteworthy that in contrast to the harmful TLR overactivation in circulating neutrophils, adequate activation of TLRs in migrated neutrophils is crucial for establishing the local immune response. Indeed, *tlr4*-mutant mice fail to control a low-dose infection with the Gram-negative bacterium *Salmonella typhimurium* (36). Moreover, *myd88*-deficient mice are highly susceptible to polymicrobial sepsis because the lack of the adaptor protein involved in most of TLR signaling prevents the establishment of the local inflammatory response. In contrast to the TLRs, the pattern recognition receptors Nod-like receptors 1 and 2 are not involved in neutrophil migration to the site of infection or in the establishment of the inflammatory response locale in mice subjected to CLP-induced polymicrobial sepsis (38).

Further investigation of how TLRs modulate the expression of CXCR2 on the neutrophil surface suggested the involvement of tumor necrosis factor (TNF)-α and NO. Neutrophils isolated from *tnf receptor*-deficient mice activated with LPS do not show internalization of CXCR2 or impaired chemotaxis to CXCL2. Additionally, neutrophils treated with TNF-α exhibit reduced chemotaxis toward CXCL2 (27). Moreover, NO confers a similar effect in LPS- or IL-8-stimulated neutrophils. Indeed, inhibition of iNOS reduces the effect of the LPS or IL-8 on the internalization of CXCR2 and the chemotactic activity of CXCR2 agonists (24, 29). NO triggers the activation of soluble guanylate cyclases (GCs) as well as cyclic-GMP formation and protein kinase G (PKG) phosphorylation (39). As expected, inhibition of sGC or PKG had the same effect as iNOS inhibition after LPS stimulation of neutrophils. Interestingly, inhibition of sGC and PKG during experimental sepsis protected mice from death, and this effect was associated with reduced expression of GRK2 in neutrophils, increased expression of CXCR2 and, consequently, increased neutrophil migration to the infectious focus compared with non-treated animals (29).

Based on these observations, we could suggest that TNF-α production and/or release in neutrophils is important to the effect of TLRs on the CXCR2 expression on these cells. In addition, both TLR- and TNF-dependent pathways upregulate inducible NO synthase, which could in turn induce GRK2 expression, leading to reduced CXCR2 expression on the ­neutrophil surface (40).

In addition to TNF-α and iNOS, it has been demonstrated that phosphoinositide-3 kinase gamma (PI3Kγ) plays an important role in this process (28). Interestingly, PI3K may be involved in the dimerization of iNOS, an essential process for the activity of this enzyme (41). GRK2 upregulation and CXCR2 internalization were shown to be inhibited in *PI3K*γ^−/−^ neutrophils incubated with CXCL2. Additionally, *PI3K*γ^−/−^ mice subjected to CLP present reduced GRK2 expression and increased CXCR2 expression on the neutrophil surface, resulting in higher survival rates (28). Altogether, these data provide substantial evidence of the links between all pathways discussed above and highlight new potential targets for sepsis treatment.

In contrast to the deleterious role of the pathways described above, there are also mediators that protect the organism against sepsis. One example is hydrogen sulfide (H_2_S), a gas produced by the organism that is synthesized from l-cysteine, mainly *via* the cystathionine b-synthase and cystathionine g-lyase (CSE) enzymes (42). It has been demonstrated that CSE activity is increased during sepsis, and inhibition of CSE reduces CLP-induced leukocyte–endothelial interactions in mesenteric venules, decreases neutrophil migration to the site of infectious, and consequently decreases the survival rate of animals subjected to non-severe sepsis (43). In contrast, treatment of mice subjected to severe sepsis with an H_2_S donor has the opposite effect, resulting in increased CXCR2 expression on circulating neutrophils, increased neutrophil migration to the infection focus, and improvement survival (43). Thus, H_2_S donors could be considered for use in sepsis treatment.

Additionally, neutrophil migration events during sepsis have been demonstrated to be regulated by several other mediators, such as lectin-like oxidized low-density lipoprotein receptor (LOX)-1, peroxynitrite, and the acute-phase alpha-1 acid protein, which contribute to the failure of neutrophil migration to the site of infection (44–46). Conversely, the cytokine IL-17 has been shown to be crucial for recruiting neutrophils to the site of infection during sepsis (47). In contrast, the role of the peroxisome proliferator-activated receptor in neutrophil migration during sepsis remains to be confirmed, as both protective and deleterious role have been described (48, 49). The effects of these mediators on the neutrophil migration have been reviewed elsewhere (20, 50, 51) and will not be further addressed here.

## Neutrophil-Induced Organ Damage

In addition to the host-protective role of neutrophils in sepsis *via* the killing of microorganisms, these cells have been described as exhibiting deleterious functions (6). During sepsis, it has been shown that the systemic inflammatory response leads to the activation of circulating neutrophils sequestered in capillary beds, occluding the lumen, and inducing tissue ischemia. Additionally, neutrophils can migrate to vital organs and release lytic factors and pro-inflammatory cytokines, contributing to organ damage and subsequent multiple organ dysfunction (52, 53). Chemokines and chemokine receptors are also involved in the process of neutrophil infiltration into vital organs during sepsis. In contrast to the observation that CXCR2 is internalized in circulating ­neutrophils during severe sepsis (24), this receptor has been implicated in neutrophil infiltration into the lungs, due to the release of CXC chemokines in this organ during sepsis (54, 55). This apparent contradiction could be explained by the differences in the severity of sepsis induced in each study.

Furthermore, our group and others have demonstrated that CCR receptors, which are not expressed on neutrophils under physiological conditions, are induced in this cell type in various inflammatory processes (56–60). It was demonstrated that CCR2 is induced on the neutrophil surface in mice and patients with sepsis in a TLR2- or TLR4-dependent manner. Importantly, CCR2 does not mediate neutrophil recruitment to the site of infection, but it does mediate neutrophil infiltration in vital organs, such as the lungs, kidneys, and heart. Blockage of CCR2 decreases organ damage and death in animals subjected to severe CLP. Moreover, CCR2 expression is positively correlated with the severity of the disease, as measured using the Sepsis-related Organ Failure Assessment (SOFA) score. Accordingly, human neutrophils isolated from non-surviving septic patients express more CCR2 than neutrophils from surviving patients (59).

Another important feature of neutrophils is the formation of NETs, a network of chromatin fibers associated with granules of antimicrobial peptides and enzymes such as myeloperoxidase, elastase, and cathepsin G, which immobilize and kill invading microorganisms to prevent their spreading (61). The role of NETs in the control of bacterial spreading in sepsis is controversial. Similar bacterial loads were observed in animals lacking an important enzyme (peptidylarginine deiminase 4) for NET formation and in animals treated with rhDNAse compared with control mice (62, 63). However, our group and others (64, 65) have observed an important role of NETs in the control of bacterial spreading during sepsis.

In addition to the role of the NETs in bacterial control during infection, excessive formation of NETs has been observed in many pathological conditions, which is related to organ damage (66). Activated endothelial cells induce the formation of NETs by neutrophils *in vitro* (67, 68). Moreover, in an LPS-induced endotoxic shock model, NETs have adhered and activated the vascular endothelium (69). Additionally, the interaction between neutrophils and activated platelets during sepsis induces NET formation, which contributes to endothelial cell damage and organ injuries (70). Moreover, it has been reported that histones and myeloperoxidase could be responsible for NET-induced endothelial dysfunction, and histones can also interact with TLR2 and TLR4 to induce cytokine production *via* MyD88 signaling, contributing to the systemic inflammatory response observed in sepsis (68, 71–74).

Surprisingly, the survival rate was not found to differ between rhDNAse-treated and non-treated mice after CLP (65). Further investigations revealed that this lack of a difference was due to the deleterious role of NETs in organ damage, as discussed above. Thus, when antibiotic therapy and rhDNAse treatment or inhibition of the enzyme peptidylarginine deiminase 4 were used in combination to control a bacterial infection, a marked increase in the survival rate of the animals was observed, which was associated with decreased organ damage (63, 65). In ­addition, pretreatment with rh-DNAse in animals challenged with LPS decreased the organ damage and increased the survival rate during endotoxemia (65).

The observations from mouse models confirm the human ones. Notably, autopsy examinations of tissues from septic patients with multiple organ dysfunction syndrome indicate the presence of neutrophils sequestered into the kidneys and lungs (75). Furthermore, severity of acute respiratory distress syndrome in septic patients is proportional to the intensity of the inflammatory infiltrate and proteolytic enzymes in the bronchoalveolar lavage (76).

During sepsis, organ failure is associated with hypoperfusion and tissue hypoxia, both of which are attributed to hypotension and occlusion of neutrophils in the microcirculation (77, 78). The cytokines secreted by neutrophils attached to a vessel wall can also cause endothelial dysfunction, establishing a thrombogenic profile and favoring intravascular coagulation (79). Additionally, neutrophil products can also induce increased NO production by various cell types, which can contribute to lowering blood pressure (80) and favors the generation of peroxynitrite, a potent oxidant agent. In the heart, peroxynitrite can cause changes in the structure and function of proteins that may be related to sepsis-associated myocardial failure (81). Thus, the adhesion of neutrophil to the endothelium and their sequestration to the heart may have multiple deleterious cardiovascular effects.

## Conclusion and Perspectives

It is clear that sepsis continues to represent a challenge for basic and clinical researchers. Despite the massive amount of basic and clinical results related to this syndrome that has been published in the literature in the last several decades, there has been an absence of effective new treatments. The high mortality associated with sepsis together with its increased incidence, points to the importance of re-evaluation of the literature as well as the new translational studies addressing the disease. Together, these approaches will help to identify new effective targets for the development of new therapies. In this context, the present review described the dual roles of neutrophils in the evolution of sepsis. These cells are key players in the innate immune response in the early phase of sepsis and their recruitment to sites of infection is crucial for controlling microorganism growth. Aggravation of sepsis is associated with failure of neutrophil migration to the site of infection. The molecular mechanism involved in this phenomenon was described, and several potential targets for the development of new therapies were identified. By contrast, neutrophils can be harmful and induce secondary organ damage during infection. Neutrophil recruitment to organs far from the site of infection is mediated by the expression of CCR2 under septic conditions. The mechanism involved in the harmful effect of the neutrophils was also described in this review, noting potential targets for the development of new therapies. In this context, new therapies targeting the harmful activity of neutrophils, such as blocking NET formation or CCR2 activity, might be more helpful than targeting the general inflammatory response.

## Author Contributions

FS, FC, AK, CL, RF, DN, DC, VB, JA-F, and FC wrote and approved the text. CL draws the figure.

## Conflict of Interest Statement

The authors declare the absence of any commercial or financial relationships that could be construed as a potential conflict of interest.
